# RFID-ExSim: A multi-scenario experimental dataset for collision timing, tag cloning, replay injection, and flooding stress in passive RFID systems

**DOI:** 10.1016/j.dib.2026.112575

**Published:** 2026-02-12

**Authors:** Yasser Hmimou, Mohammed Boutlane, Mohamed Tabaa, Azeddine Khiat, Zineb Hidila

**Affiliations:** a Multidisciplinary Laboratory of Research and Innovation (LPRI), Moroccan School of Engineering Sciences (EMSI), Casablanca 20250, Morocco; b 2IACS Laboratory, ENSET, Hassan II University of Casablanca, Casablanca 20360, Morocco

**Keywords:** RFID security dataset, Collision timing, UID cloning, Replay injection, Flooding stress, Intrusion detection, IoT authentication

## Abstract

RFID-ExSim is an experimental dataset designed for studying passive RFID systems under normal operating conditions and in adversarial attack scenarios. The dataset was collected in a controlled laboratory environment using two synchronized ESP32-based MFRC522 RFID readers interacting with twelve passive RFID tags. In total, >400,000 raw RFID read events were recorded and stored in a structured JSONL format with millisecond-level timestamp resolution.

The dataset is structured around five well-defined acquisition scenarios: (i) basic single-reader operation, (ii) dual-reader collision interference, (iii) tag cloning at the UID level, (iv) replay and software injection attacks, and (v) high-throughput stress sessions simulating denial-of-service conditions. Each recorded event includes the reader ID, local tag ID, hashed UID, raw payload, scenario label, and physical acquisition parameters, including tag-to-reader distance and angular orientation.

RFID-ExSim offers a reproducible and fully documented benchmark for analyzing RFID reliability, collision dynamics, identity cloning, replay behavior, and system robustness under high load conditions. The dataset is designed to support research in the areas of RFID security, physical layer analysis, anomaly detection, intrusion detection systems, and machine learning-based RFID authentication.

Specifications Table

**Table:** 

Subject	Computer Sciences
Specific subject area	Passive RFID security experiments and attack scenario simulation
Type of data	Raw, Processed, Raw experimental logs in JSON Lines (JSONL) format.
Data collection	The dataset was collected using a controlled testbed featuring two synchronized ESP32 microcontrollers and MFRC522 RFID readers (13.56 MHz). Data were captured from twelve passive RFID tags under five scenarios (Baseline, Collision, Cloning, Replay, and Flooding) with variable distance (1, 3, 4 cm) and orientation (0°, 45°, 90°). Events were logged with millisecond-level timestamp resolution.
Data source location	Multidisciplinary Laboratory of Research and Innovation (LPRI), Moroccan School of Engineering Sciences (EMSI), Casablanca 20,250, Morocco
Data accessibility	Repository name: Zenodo Data identification number (or DOI or persistent identifier): DOI: 10.5281/zenodo.17854328 Direct URL to data: https://doi.org/10.5281/zenodo.17854328 Instructions for accessing these data: All RFID-ExSim dataset files (raw JSONL logs, consolidated per-scenario datasets, metadata, and experimental plans) are publicly available without access restrictions via the Zenodo repository. The accompanying codebase, including ESP32 firmware and analysis scripts, is openly accessible on GitHub at: https://github.com/yasserhmimou9/RFID-ExSim-Dataset
Related research article	‘none’

## 1 Value of the Data

• The RFID-ExSim dataset provides a large-scale, structured collection of more than 400,000 real passive RFID read events acquired under controlled baseline and adversarial conditions. It includes five well-defined scenarios covering single-reader operation, multi-reader collisions, UID cloning, replay injection, and flooding stress. This diversity allows researchers to study both nominal RFID behavior and security-relevant threat patterns under reproducible experimental settings.
• The dataset can be reused to benchmark collision detection, replay attack identification, and RFID-based intrusion detection algorithms. The availability of synchronized dual-reader logs with millisecond-resolution timestamps enables precise temporal analysis of interference, desynchronization, and UID conflict events across multiple physical configurations.
• The structured JSONL format with rich metadata (distance, orientation, scenario ID, tag ID, reader ID, timestamp) facilitates direct reuse for machine learning, anomaly detection, and physical-layer authentication research. Researchers can directly train and evaluate supervised and unsupervised models without additional data cleaning or reconstruction.
• The inclusion of UID cloning and replay injection scenarios provides empirical traces of identity-based attacks that are rarely available in open RFID datasets. These traces enable systematic testing of clone detection strategies, fingerprinting methods, and replay-resistant authentication mechanisms under real acquisition conditions.
• The flooding stress scenario offers high-rate RFID traffic suitable for denial-of-service modeling and resilience evaluation. The associated logs expose read saturation, success-rate degradation, and reader asymmetry effects, which can be reused for time-series analysis, congestion modeling, and stress testing of RFID security systems.

## 2 Background

RFID systems are deployed widely in industrial logistics, access control, healthcare traceability, and IoT infrastructure due to their passive operation, low power consumption, and interoperability with embedded platforms [1]. However, their growing adoption has also exposed them to multiple security threats, including unauthorized identification, tag spoofing, unique identifier cloning, replay attacks, and communication flooding [2,5]. Previous [3,4] studies have demonstrated that passive RFID tags can be reverse engineered, cloned at low cost, and exploited by manipulating backscatter signals based on replay [2,6], but most of this work relies on limited or proprietary experimental data.

Recent research has explored physical layer attributes and temporal behavior as potential discriminators for clone detection and anomaly classification [7–11]. These [10] approaches require synchronized measurements, collision observations, and controlled adverse conditions, but the lack of open, structured, and reproducible RFID datasets remains a major limitation for comparative evaluation [12,13]. In particular, existing studies rarely provide empirical data covering collisions between multiple readers, replay injection, UID-level cloning, and high-load stress conditions under controlled geometric parameters [3,6,8,9].

The RFID-ExSim dataset was constructed to address this empirical gap through systematic acquisition of passive RFID interactions under nominal and adversarial scenarios [14]. By recording synchronized multi-reader events with controlled distance and orientation parameters, the dataset provides a unified experimental basis for studying RFID timing behavior, collision dynamics, identity duplication, and replay-induced artifacts.

## 3 Data Description

The RFID-ExSim dataset is distributed through a public repository and organized into structured directories containing raw acquisition logs, consolidated scenario datasets, metadata files, experimental plans, derived summary files, firmware source code, and documentation. All files are provided to enable direct access to the complete experimental recordings.

### 3.1 Main repository structure

The dataset is organized into the following main directories:

• data/
• docs/
• figures/
• firmware/
• tags.csv

### 3.2 Raw data directory

The folder data/raw/ contains the complete set of original RFID acquisition logs recorded directly from the two ESP32–MFRC522 readers. Each acquisition run is stored as two synchronized JSON Lines (.jsonl) files, one for each reader:

• session_ <Scenario> _<Tag>_<run>_ ESP32_A.jsonl
• session_ <Scenario> _<Tag>_<run>_ ESP32_B.jsonl

Each raw file contains one JSON object per line corresponding to a single RFID read event. Each event includes the following attributes:

• timestamp (millisecond resolution)
• reader_id
• uid_hash
• payload
• tag_local_id
• scenario
• session_id
• distance_cm
• orientation_deg

For each session, an associated .meta file is provided containing the experimental parameter settings.

### 3.3 Processed scenario files

The directory data/processed/ contains five consolidated JSONL files, each grouping all acquisition sessions for one experimental scenario:

• rfid_dataset_S1_all.jsonl
• rfid_dataset_S2_all.jsonl
• rfid_dataset_S3_all.jsonl
• rfid_dataset_S4_all.jsonl
• rfid_dataset_S5_all.jsonl

These files contain the same event structure as the raw logs and combine all runs related to each scenario.

### 3.4 Scenario execution plans

The directory data/plans/ contains five CSV files defining the acquisition parameters for each scenario:

• s1_plan.csv
• s2_plan.csv
• s3_plan.csv
• s4_plan.csv
• s5_plan.csv

Each plan file specifies:

• session_id
• tag_local_id
• distance_cm
• orientation_deg
• number_of_repetitions

### 3.5 Calibration data

The directory data/calibration/ contains calibration measurements used for baseline configuration of the RFID system prior to scenario execution.

### 3.6 Metadata and summary files

The dataset includes structured metadata and summary descriptors derived from the raw logs:

• meta_sessions_S1.csv
• meta_sessions_S2.csv
• s2_collisions_summary.csv
• s3_clone_collisions_summary.csv
• s3_summary_counts.csv
• s4_merge_summary.json
• s4_replay_summary.csv
• s5_success_rates_per_session.csv

These files provide per-session and per-scenario descriptors without modifying the original raw events.

### 3.7 Documentation files

The folder docs/ contains dataset documentation:

• methodology.md: describes acquisition procedures and logging formats
• scenarios.md: defines the five experimental scenarios and execution rules

### 3.8 Figures directory

The figures/ directory contains graphical visualizations derived directly from the dataset, including:

• read distributions
• collision histograms
• replay latency distributions
• flooding scenario time series
• read asymmetry plots

Each figure is associated with one specific scenario.

### 3.9 Firmware directory

The directory firmware/ contains the firmware used for RFID acquisition:

• **esp32_reader/**: source code for the ESP32 reader nodes
• notes_wiring.md: hardware wiring configuration notes

### 3.10 Tag mapping file

The file tags.csv maps the physical RFID tags to anonymized identifiers. The file includes:

• tag_local_id
• uid_hash
• initial_label

### 3.11 Dataset overview

• Number of RFID readers: 2
• Number of RFID tags: 12
• Number of scenarios: 5
• Data format: JSON Lines (JSONL), CSV
• Timestamp resolution: millisecond
• Total number of records: over 400,000 RFID read events
• Spatial acquisition parameters: distance (1, 3, 4 cm), orientation (0°, 45°, 90°)

## 4 Experimental Design, Materials and Methods

**Fig. 1:**
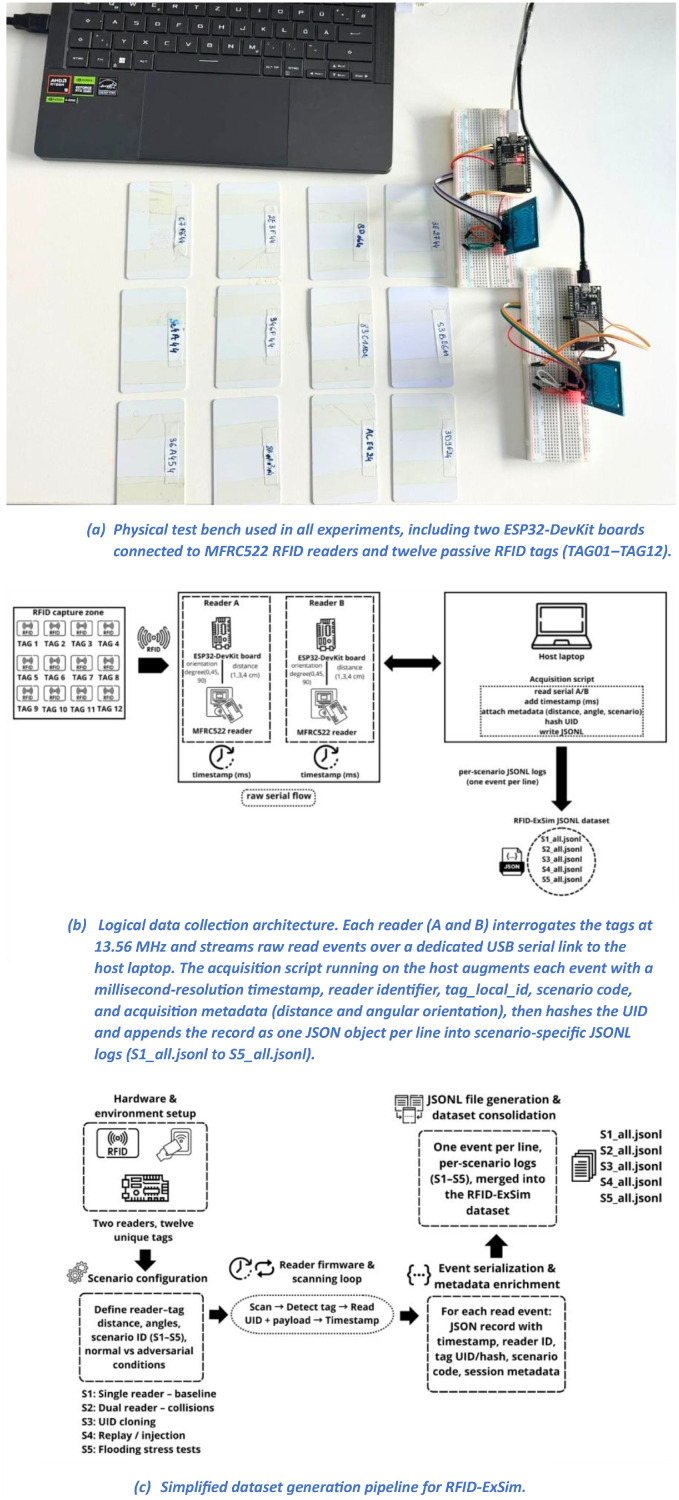
Experimental setup and dataset generation pipeline for RFID-ExSim.

The RFID-ExSim dataset was acquired using a controlled experimental testbed designed to ensure repeatable and synchronized data acquisition across multiple RFID attack and stress scenarios. The acquisition system is composed of two ESP32-DevKit microcontroller boards, each connected to an MFRC522 RFID reader operating at 13.56 MHz. The two readers are designated as Reader A and Reader B and are connected via USB serial links to a host laptop used for centralized data logging. Twelve passive RFID tags compliant with ISO/IEC 14443-A were used throughout all experiments. The physical experimental testbed, including the two ESP32-DevKit boards, MFRC522 RFID readers, and passive tags, is illustrated in Fig. 1.a.

Each ESP32 executes identical firmware developed using the Arduino framework. The firmware continuously scans for nearby RFID tags using a polling loop and transmits each successful detection event over the serial interface. For each detection, the firmware outputs the raw UID, reader identifier, and the exact acquisition timestamp. On the host machine, a Python-based acquisition program records all incoming serial events from both readers in real time and converts them into structured JSONL (JSON Lines) files. The logical acquisition architecture and real-time data flow from RFID detection to structured JSONL logging are depicted in Fig. 1.b. Each recorded event includes the following fields: timestamp (millisecond resolution), reader ID, local tag ID, hashed UID, raw payload, scenario label, session ID, tag-to-reader distance (cm), and tag angular orientation (degrees).

The ESP32 firmware was developed and compiled using Arduino IDE version 1.8.19. RFID communication was implemented using the MFRC522 Arduino library version 1.4.12 (SPI interface), compatible with ISO/IEC 14443-A tags. On the host machine, the acquisition pipeline was implemented in Python 3.10 using pyserial 3.5 for serial communication, hashlib for UID hashing, and json for JSONL serialization. All software dependencies, firmware source code, and acquisition scripts are publicly available in the associated GitHub repository and documented in the methodology.md file.

All experiments were conducted under fixed geometric and environmental conditions. The tags were placed on a non-metallic surface to avoid electromagnetic interference. Three discrete distances between tag and reader were used: 1 cm, 3 cm, and 4 cm. Three angular orientations were also applied for each tag: 0°, 45°, and 90° These physical parameters were manually controlled and systematically varied across all acquisition sessions.

Data acquisition is organized into five distinct experimental scenarios. Scenario S1 corresponds to baseline single-reader operation, where only one reader actively scans tags without interference. Scenario S2 implements dual-reader simultaneous operation to induce collision conditions. Scenario S3 reproduces tag cloning through UID duplication, where multiple tags broadcast identical identifiers. Scenario S4 implements replay and software-based injection attacks by reinjecting previously recorded UIDs through controlled firmware-level emulation. Scenario S5 generates flooding and high-throughput stress conditions by forcing continuous repeated UID transmissions to simulate denial-of-service behavior. An overview of the RFID-ExSim dataset generation pipeline and the five experimental scenarios is shown in Fig. 1.c.

Each scenario is executed in multiple acquisition sessions. For every session, the corresponding scenario label, physical distance, and orientation are explicitly encoded in the dataset metadata. The flood and cloning scenarios are generated through controlled manipulation of the firmware and the Python acquisition scripts. No synthetic data generation or simulation-based modification is applied; all records correspond to physical acquisitions captured directly from the hardware setup.

The final dataset structure consists of (i) raw JSONL logs separated by scenario and session, (ii) merged per-scenario datasets aggregating all sessions, (iii) metadata files describing tag identities, reader assignments, distance and orientation parameters, and (iv) experimental configuration files documenting acquisition conditions. All firmware source code for the ESP32 readers, along with the Python acquisition and parsing scripts used for dataset generation, are made publicly available in the associated GitHub repository.

## Limitations

The dataset was acquired in a controlled indoor laboratory environment using a fixed experimental configuration composed of two ESP32-based MFRC522 RFID readers and twelve passive RFID tags. Consequently, the environmental conditions such as temperature variability, electromagnetic noise from external sources, and large-scale spatial deployment were not explored.

The acquisition parameters were defined using three discrete tag-to-reader distances (1 cm, 3 cm, and 4 cm) and three angular orientations (0°, 45°, and 90°), which represent a subset of possible geometric configurations.

All experiments were conducted using a single RFID technology operating at 13.56 MHz and identical reader hardware and firmware versions. The dataset does not include additional RFID standards, frequency bands, or reader models.

These elements define the scope of the experimental acquisition conditions without affecting the integrity or reproducibility of the recorded data.

## Ethics Statement

The authors confirm that they have read and comply with the ethical requirements for publication in Data in Brief. The current work does not involve human subjects, animal experiments, or any data collected from social media platforms. The dataset was generated exclusively using RFID hardware devices under controlled laboratory conditions, without involving personal data, biological materials, or human participation.

## CRediT authorship contribution statement

**Yasser Hmimou:** Conceptualization, Methodology, Software, Investigation, Data curation, Formal analysis, Writing – original draft. **Mohammed Boutlane:** Methodology, Validation, Resources, Writing – review & editing. **Mohamed Tabaa:** Supervision, Conceptualization, Project administration, Writing – review & editing. **Azeddine Khiat:** Supervision, Writing – review & editing. **Zineb Hidila:** Supervision, Writing – review & editing.

## Data Availability

zenodoRFID-ExSim: An Experimental Dataset for Passive RFID Security Under Collision, Cloning, Replay, and Stress Conditions (Original data).
